# An RNA-sequencing analysis to determine potential upstream transcriptional regulators of essential amino acid deficiency responses in bovine mammary epithelial cells

**DOI:** 10.1186/s12864-026-12791-y

**Published:** 2026-03-27

**Authors:** Boning Li, Dave J. Innes, Eduardo S. Ribeiro, John Doelman, Sergio A. Burgos, John P. Cant

**Affiliations:** 1 Department of Animal Biosciences, University of Guelph, Guelph, ON Canada; 2 School of Environmental and Rural Science, University of New England, Armidale, Australia; 3 Trouw Nutrition R&D, Amersfoort, the Netherlands; 4 Department of Animal Science, Faculty of Agriculture and Environmental Science, McGill University, Quebec City, QC Canada

**Keywords:** Essential amino acid deficiency, Transcription factors, Cell cycle progression, Interferon signaling, ATF4, FOXM1, Bovine mammary epithelial cell

## Abstract

Essential amino acids (EAA) may alter bovine mammary epithelial cell (BMEC) activities through regulation of gene transcription. For this study, we used pathway analysis of RNA-sequencing data to identify upstream transcription factors (TF) that potentially mediate the effects of single EAA deficiency on BMEC. Differentiated BMEC were cultured in 1 of 4 treatment media representing normal physiological concentrations of all amino acids (CTL), or ¼ the normal concentration of histidine (LH), lysine (LK), or methionine (LM). After 48 h, rates of DNA synthesis were reduced 30 to 40% by each of the deficiencies. Protein synthesis rate was 30 to 50% lower in LK and LM but was unaffected by LH. Up-regulated upstream TF included those related to nutrient stress, interferon signaling, and suppression of cell population growth. Down-regulated TF were primarily related to cell cycle progression. An array of classic ATF4 targets increased in response to EAA deficiencies including amino acid transporters, branched-chain aminotransferase, aminoacyl-tRNA synthetases, sestrin2 (a leucine sensor), and activators of apoptosis. Interferon signaling emerged as a novel pathway activated by EAA deficiencies, possibly contributing to reduced cell proliferation and protein synthesis. In contrast, the MAPK signaling pathway was unaffected. Expression of hypoxia-inducible factor 1α (HIF1A) increased, consistent with an anti-proliferative role. Downregulation of Forkhead box protein M1 (FOXM1) likely contributed to slower cell proliferation. These findings reveal a complex network of transcriptional regulators and signaling pathways influenced by EAA availability and shed light on the nutritional regulation of milk protein production by dairy cows.

**Supplementary Information:**

The online version contains supplementary material available at 10.1186/s12864-026-12791-y.

## Background

 The intakes of dietary energy and essential amino acids (EAA) are well documented as major determinants of milk component yields in lactating dairy cows [1, 2]. However, the intracellular mechanisms by which supplementation of dietary nutrients, particularly EAA, stimulates milk and milk protein production are not completely understood. EAA are not only building blocks of proteins but also serve the function of signaling mediators in several regulatory pathways in mammary cells, including the mammalian target of rapamycin complex 1 (mTORC1) and eukaryotic initiation factor 2α (eIF2 α) kinase networks [3, 4]. These pathways are a means by which EAA rapidly affect the global mRNA translation rate, within minutes or hours. However, the longer-term transcriptional response to EAA supply remains poorly characterized.

Sustained nutritional effects on milk component yields are mediated through alterations in mammary epithelial cell mass, driven by modifications in protein synthesis, cell proliferation, differentiation and apoptosis [5]. Transcription factors (TFs) that regulate gene transcription have been studied as mediators of cell proliferation and apoptosis responses to nutrition in many species and tissues. We previously hypothesized a set of eight TFs involved in communicating AA status to bovine mammary epithelial cells (BMEC) and found that Activating transcription factor 4 (ATF4) was activated by lysine and methionine deprivation [6]. ATF4 is the classic AA response mediator in cells that is activated by general control nonderepressible 2 (GCN2) during EAA deficiency to respond to and compensate for the stress [7]. The compensation is achieved by inducing expression of genes for AA transporters such as cationic AA transporter (SLC7A1) and branched chain AA transporter (SLC7A5), and for AA synthesizing enzymes such as asparagine synthetase (ASNS) ([7, 8]. Simultaneously, ATF4 suppresses the mTORC1 pathway of cell growth promotion through DNA damage-inducible transcript 4 (DDIT4) and sestrin2 (SESN2) [9], and promotes cell apoptosis through DDIT3 [7, 8]. It appears that ATF4 regulates complex signaling pathways to counter cellular stress by increasing the availability of AA within cells, inhibiting protein synthesis, and promoting apoptosis.

Another potential means of transcriptional regulation in response to EAA is through the MAPK signaling pathway of RAS/RAF/MEK/ERK [10]. However, our previous research found that MAPK target genes FOS and JUN were not activated in BMEC by more than 24 h of lysine and methionine deficiency [6]. Instead, we found that the TFs hypoxia-inducible factor-1 alpha subunit (HIF1A) and early growth response 1 (EGR1) were respectively up- and down-regulated by methionine deficiency in accord with the slower proliferation rate [6]. Lysine deficiency did not produce these effects. Evidence indicates that EAA deficiency may influence cell proliferation through the ATF4 or MAPK pathways or other unidentified pathways. Furthermore, there may be undiscovered TFs involved in an EAA stress response in BMEC. Investigating these potential TFs within the signaling network is crucial to advancing our understanding of the regulatory effects of EAA in the mammary glands of dairy cows.

To identify potentially novel TFs that mediate the effects of EAA deficiency on mammary cell activity, we used a genome-wide approach based on RNA-sequencing (RNA-Seq) of gene expression in BMEC exposed to histidine, lysine, or methionine deprivation for 60 h. Deficiencies in exogenous supply of each of these EAA have been found to depress daily milk protein yields from the mammary glands of lactating cows [11, 12]. Differentially expressed genes (DEG) were detected to characterize BMEC responses, but the data were primarily used to identify key upstream transcriptional regulators of the responses to EAA deficiency.

## Materials and methods

### Isolation and culture of bovine mammary epithelial cells

Details of the isolation and culture of BMEC were described in Li et al. [6]. Briefly, mammary tissue was collected from lactating Holstein cows post-slaughter. The tissue was cleaned, minced, and digested with collagenase Type III and DNase I at 37 °C for 4 h. After filtration and centrifugation, the enriched epithelial organoids were either cryopreserved or seeded in collagen-coated dishes with a specialized medium. The culture medium consisted of a 1:1 mixture of DMEM/F12 and MCDB170, supplemented with 10% (vol/vol) fetal bovine serum, 0.1% (wt/vol) albumax II, 7.5 µg/mL bovine insulin, 0.3 µg/mL hydrocortisone, 5 ng/mL recombinant human epidermal growth factor, 2.5 µg/mL bovine apo-transferrin, 5 µM isoproterenol, 5 pM 3,3′,5-triiodo-l-thyronine, 0.5 pM β-estradiol, 0.1 nM oxytocin, and 1× antibiotics/antimycotics. Medium was refreshed every 3 d during the first week and, once most organoids had attached to the dish, replaced every 2 d in an incubator at 37 °C with a 5% CO_2_ humidified atmosphere until primary mammary epithelial cells were further passaged.

All experiments were performed using third passage BMEC. Cells were seeded on 6-well cell culture plates and grown to confluence in a medium containing 0.25% fetal bovine serum at 37 °C with a 5% CO_2_ humidified atmosphere. The BMEC were differentiated for 5 d in DMEM modified to contain 3.5 mM D-glucose, 2 mM sodium acetate, and 200 µM L-glutamine, supplemented with lactogenic hormones (5 µg/mL each of bovine insulin, prolactin, and hydrocortisone), 5 µg/mL apo-transferrin, 0.5 mg/mL BSA, and 1× antibiotics/antimycotics. The medium was changed every 2 d.

### Treatments and sample collection

Differentiated BMEC were cultured in triplicate under four treatment conditions: normal physiological concentrations of all amino acids (CTL), low histidine (LH), low lysine (LK), or low methionine (LM) for 60 h. The base medium was DMEM containing 3.5 mM D-glucose, 2 mM sodium acetate, and 200 µM L-glutamine, supplemented with lactogenic hormones (5 µg/mL each of bovine insulin, prolactin, and hydrocortisone), 5 µg/mL apo-transferrin, 0.5 mg/mL BSA, and 1× antibiotics/antimycotics. All amino acids except lysine, methionine and histidine were added at their normal physiological concentrations based on physiological plasma AA profiles in lactating dairy cows [13, 14] (in µM): 180 L-alanine, 80 L-arginine, 50 L-Asparagine, 10 L-Aspartic acid, 12 L-Cysteine; 60 L-Glutamic acid, 170 L-Glutamine, 200 L-Glycine, 120 L-Isoleucine, 180 L-Leucine, 50 L-Phenylalanine, 70 L-Proline, 70 L-Serine, 110 L-Threonine, 30 L-Tryptophan, 50 L-Tyrosine, and 240 L-Valine. Treatments were base medium with (1) 80 µM L-Lysine, 20 µM L-Methionine, and 40 µM L-Histidine (CTL), (2) 80 µM L- Lysine, 20 µM L- Methionine, and 10 µM L- Histidine (LH), (3) 20 µM L- Lysine, 20 µM L- Methionine, and 40 µM L- Histidine (LK), and (4) 80 µM L- Lysine, 5 µM L- Methionine, and 40 µM L- Histidine (LM). After 60 h, wells were rinsed twice with phosphate-buffered saline (PBS), and cells were harvested for gene expression or protein analysis. Samples were stored at -80 °C until further analysis.

### RNA extraction

Total RNA was extracted from the cells using the Invitrogen PureLink RNA Mini Kit according to the manufacturer’s instructions. RNA concentration and purity were quantified by measuring absorbance at 260 nm and 260/280 nm, respectively, using a NanoDrop OneC microvolume spectrophotometer (Thermo Fisher Scientific, Waltham, MA). All RNA integrity numbers exceeded 8. The isolated RNA was treated with DNase to prevent DNA contamination, following the Invitrogen DNase I kit protocol.

### RNA-Seq analysis

Total RNA and library preparation quality check were performed at the Advanced Analysis Centre (University of Guelph, Guelph, Canada) using the TruSeq stranded mRNA sample preparation kit (Illumina, San Diego, USA) following the manufacturer’s protocol. RNA was sequenced on an Illumina NovaSeq 6000, producing 150 bp paired-end reads with a minimum sequencing depth of 150 million reads. Reads were cleaned with ‘fastp’ [15]. Two samples (CTL3, LM2) did not pass quality checks and were excluded from downstream analysis. Approximately 90% of the average 155 million reads per sample were aligned to the bovine reference genome (ARS-UCD1.2; NCBI: GCA_002263795.2) using ‘STAR’ aligner [16]. Genes were counted using ‘featureCounts’ [17] referenced to the Ensembl 100 annotation file (Bos_taurus.ARS-CD1.2.dna.toplevel.fa.gz).

Approximately 72% of these reads were aligned to an annotated to gene, resulting in 18,421 of 27,607 genes (67%) in the bovine reference genome with more than 10 reads across all samples. Differential expression of genes between each deficiency treatment and CTL was determined using the ‘DESeq2’ package [18] in R. Genes with an absolute log2 fold change ≥ 1 and a false discovery rate < 0.05 were considered differentially expressed. DEG were analyzed regarding their biological processes and functions and assigned to canonical pathways by Ingenuity Pathway Analysis (IPA; Qiagen Inc., www.qiagen.com/ingenuity) with default parameters in General Settings, Networks, Data sources, Confidence, Species, Tissues & Cell Lines, and Mutation. Principal component analysis (PCA) was generated by ‘DESeq2’ package in R to visualise the transcriptomic variation among samples. Upstream regulator analysis was conducted using IPA to predict the activation states of upstream regulators based on curated data from the Ingenuity^®^ Knowledge Base. IPA employs the activation z-score algorithm, which is designed to generate predictions of activation, inhibition, or no effect based on DEGs [19]. The criteria for this analysis were set to an absolute z-score ≥ 2, overlap p-value < 0.05, and molecule type = transcriptional regulator.

### Protein and DNA synthesis rate assays

Differentiated BMEC were cultured in treatment media containing either CTL, LH, LK, or LM for 60 h. Protein and DNA synthesis rates were assessed by measuring the incorporation of L-[2,3,4,5,6-³H]phenylalanine and [methyl-³H]thymidine, respectively, into an acid-precipitable fraction during the final 2 h of treatment. After 46 h of incubation, [³H]phenylalanine and [³H]thymidine were added to a final concentration of 1 µCi/mL in three separate wells for each tracer at 37 °C. Following a 2-h labeling period, wells were washed three times with PBS to stop the radioactive isotope label before cells were lysed in 0.5 mL of RIPA buffer and homogenized. DNA concentrations in 10 µL of cell lysate were determined using the DNA Qubit Assay (Invitrogen, Carlsbad, CA).

For protein and DNA precipitation, homogenates from [³H]phenylalanine and [³H]thymidine incubations were treated with 0.5 mL ice-cold 20% TCA and incubated on ice for 30 min, followed by centrifugation at 15,000 × g for 5 min. The resulting pellets were washed thrice by resuspension and centrifugation with 5% TCA. After the final wash, pellets were dissolved in 0.5 M NaOH, and 0.5 mL of the solution was mixed with 5 mL of scintillation cocktail for counting in a ³H window on a liquid scintillation counter (Beckman LS 6000, USA). Protein and DNA synthesis rates were expressed as disintegrations per minute (DPM) per nanogram of DNA.

### Statistical analysis

Observations of protein and DNA synthesis rate were subjected to one-way analysis of variance (ANOVA) by the GLIMMIX procedure of SAS Studio (SAS Institute Inc., Cary, NC, USA) with a fixed effect of treatment and random effect of replicate. Pairwise comparisons between the control and each EAA deficiency treatment were performed using Dunnett’s test. Statistical significance was declared at *P* < 0.05.

## Results

### Differential expression of genes due to single EAA deficiency reveals down-regulation of mitosis and up-regulation of interferon signaling

**Fig. 1:**
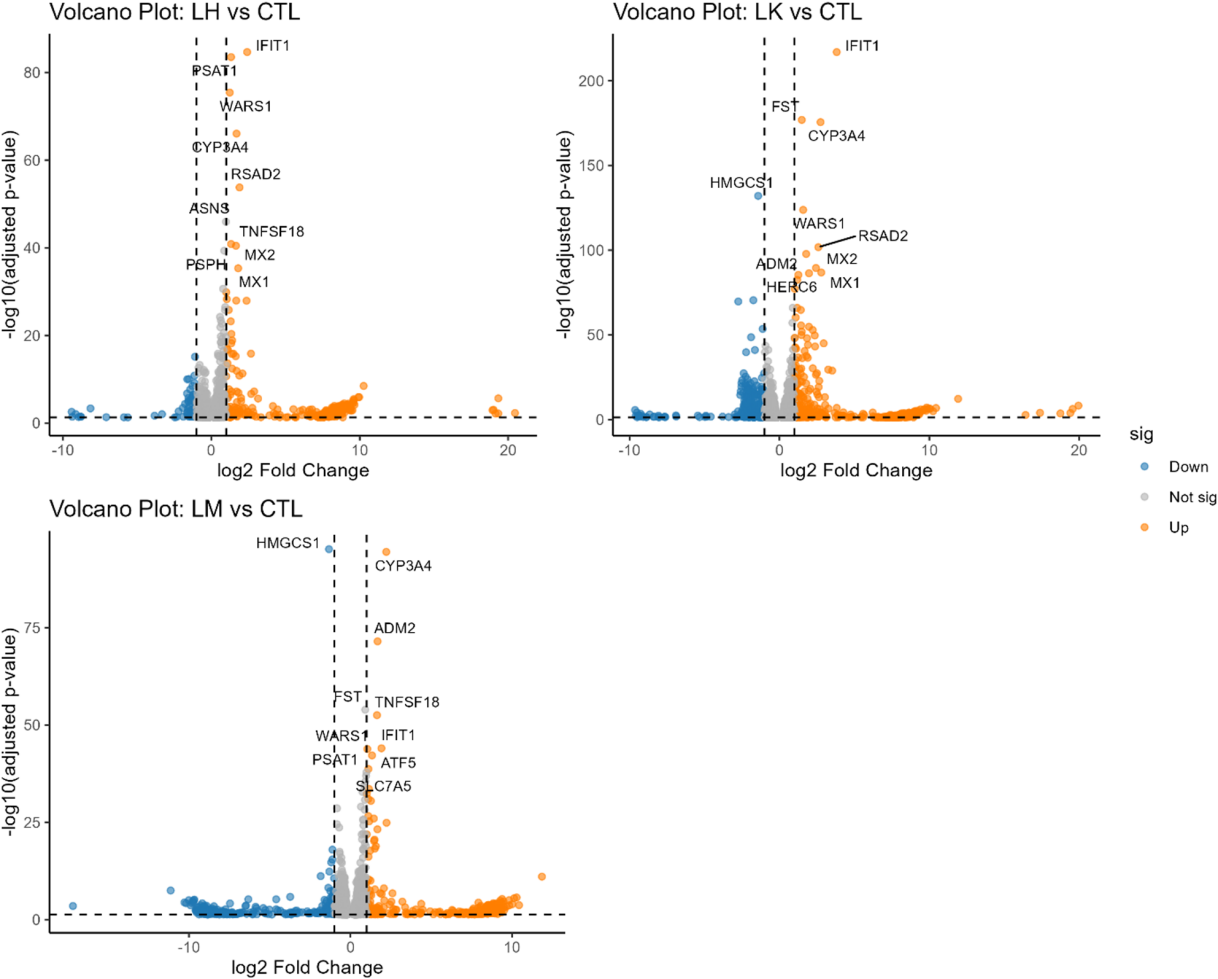
Volcano plots for differential gene expression in bovine mammary epithelial cells 60 h after histidine (LH), lysine (LK), or methionine (LM) subtraction to ¼ the normal concentration. Vertical dashed lines indicate |log₂ fold change| = 1 and the horizontal dashed line indicates FDR = 0.05. Orange and blue points represent significantly up- and down-regulated genes, respectively

**Fig. 2:**
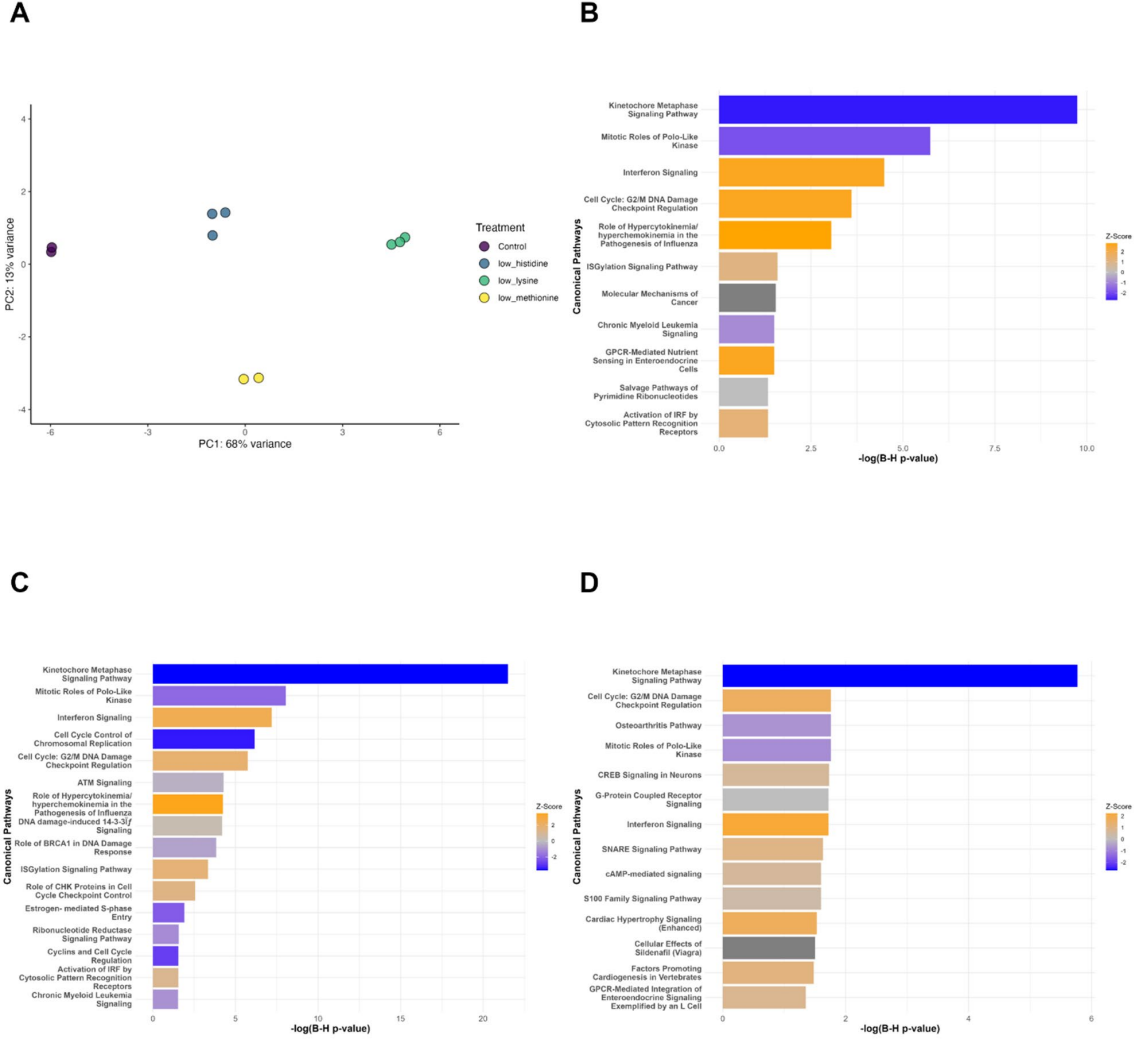
Differentially expressed genes and most significantly affected pathways from bovine mammary epithelial cells 60 h after histidine (LH), lysine (LK), or methionine (LM) subtraction to ¼ the normal concentration. The pathways were selected as *P* < 0.05. (**A**) The plot of the top 2 principal components (PC) from PC analysis of differentially expressed genes. (**B**) Most significantly affected pathways according to Ingenuity Pathway Analysis for LH treatment. (**C**) Most significantly affected pathways according to Ingenuity Pathway Analysis for LK treatment. (**D**) Most significantly affected pathways according to Ingenuity Pathway Analysis for LM treatment

Compared to CTL, the number of DEG were 383 for LH, 780 for LK, and 536 for LM when considering log2 fold change ≥ 1 or ≤ -1 and False Discovery Rate (FDR) < 0.05. DEGs between each EAA treatment and CTL were visualized using volcano plots, which illustrate the magnitude and statistical significance of gene expression changes (Fig. 1). Plots of the top 2 principal components from PCA of DEG (Fig. 2A) show a robust clustering of DEG according to treatment and a high repeatability between treatment replicates.

The highest-ranking canonical pathway enriched in DEG for each EAA deficiency treatment was the kinetochore metaphase signaling pathway, which was inhibited (Fig. 2B, C and D). Mitotic roles of polo-like kinase were also inhibited in all 3 treatments. Consistent with these proliferation-related pathway changes, the expression of hypoxia-inducible factor 1α (HIF1A), a regulator associated with anti-proliferative responses, was increased. Furthermore, LM decreased the osteoarthritis pathway, LH and LK decreased chronic myeloid leukemia signaling, and LK decreased cell cycle control of chromosomal replication, the role of BRCA1 in DNA damage response, estrogen-mediated S-phase entry, ribonucleotide reductase signaling pathway, and cyclins and cell cycle regulation. These inhibited pathways are related to cell proliferation, indicating fewer proliferating cells in EAA-deficient cultures.

Commonly up-regulated pathways across all 3 deficiency treatments were G2/M DNA damage checkpoint regulation, related to cell cycle arrest, and interferon signaling, possibly related to EAA deficiency sensing. Identification of influenza pathogenesis, ISGylation and interferon regulatory factor (IRF) activation as up-regulated pathways by IPA software is likely related to the stimulation of interferon signaling apparent in the DEG. LH and LM also activated G protein-coupled receptor pathways related to nutrient sensing, including cardiac hypertrophy signaling. LM-activated cyclic AMP-mediated signaling and transcription may be relevant to nutrient sensing.

**Fig. 3:**
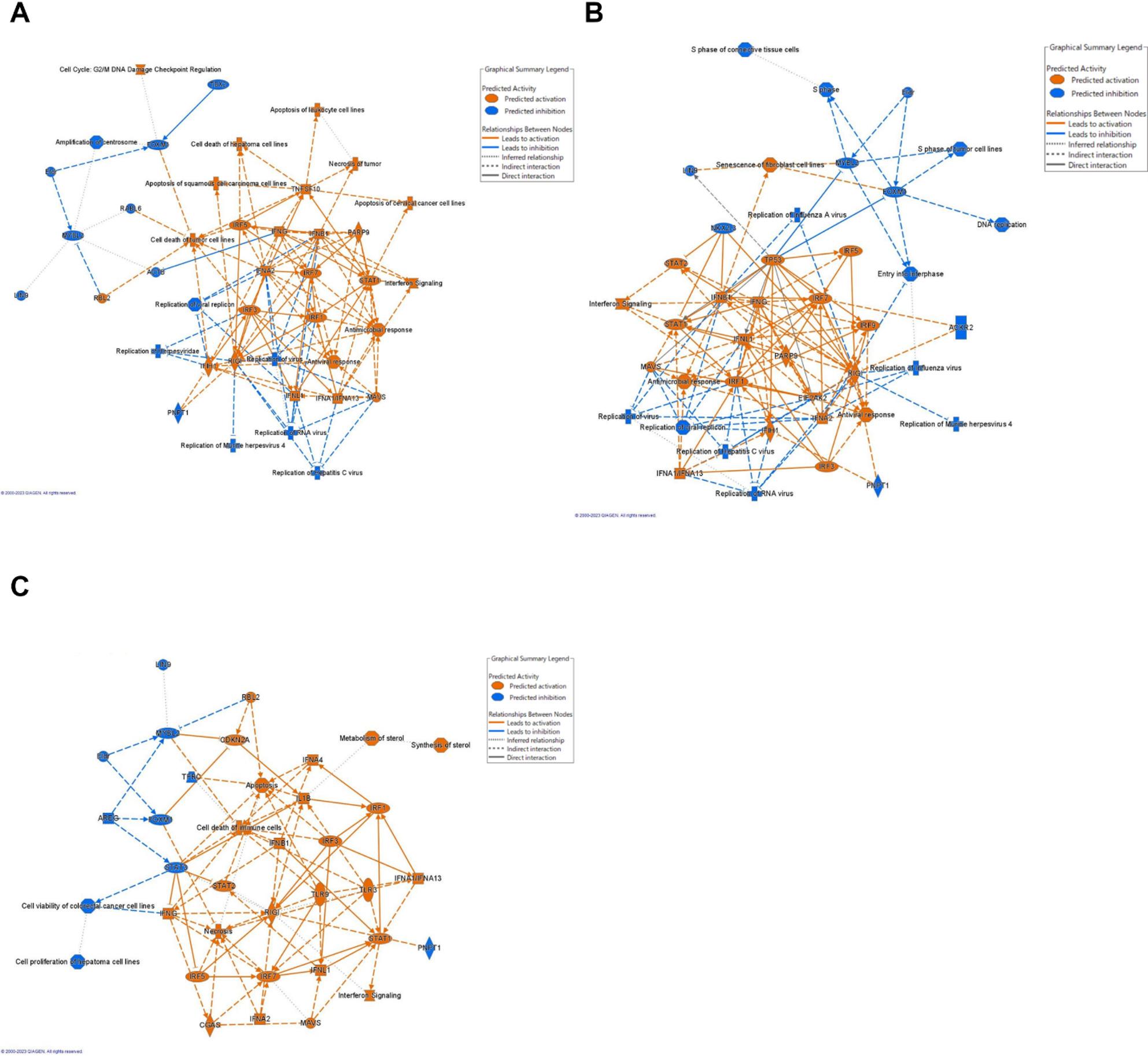
Graphical summaries of differentially expressed genes in bovine mammary epithelial cells 60 h after (**A**) histidine, (**B**) lysine, or (**C**) methionine subtraction to ¼ the normal concentration

Graphical summaries of the major EAA deficiency responses in the transcriptomes (Fig. 3) highlight the downregulation of cell proliferation, especially through the FOXM1 gene product, and the up-regulation of interferon signaling via multiple IRFs.

### Identification of TF potentially involved in mediating effects of EAA deficiency on BMEC gene expression

Based on the gene expression values in the dataset, we used IPA to generate lists of potential upstream transcriptional regulators for each treatment. According to absolute z-scores > 2, the top-ranked TF common to all three treatments are listed in Tables 1 and 2, along with the DEG supporting the identification. There were 21 TF with positive z-scores and 14 with negative scores. Up-regulated TF included those related to nutrient stress (ATF4, NUPR1), interferon signaling (IRF, SPI1, STAT), and suppression of cell population growth (CDKN2A, KAT5, PML, TCF3, TP53, TP73, TRPS1). Down-regulated TF were primarily related to cell cycle progression.

**Table 1:** Positively regulated upstream transcription regulators in bovine mammary epithelial cells 60 h after histidine (LH), lysine (LK), or methionine (LM) subtraction to ¼ the normal concentration

Upstream Regulator	Treatments	Activation z-score	Target Molecules in Dataset
ATF4	LH	3.085	ABCG8,BCAT1,CHAC1,CTH, FGF21,PSAT1,SLC1A5,SLC7A11,TRIB3,WARS1
	LK	4.022	BCAT1,CHAC1,CTH, CXCL8,FGF21,GARS1,GDF15,PCK2,PSAT1,PSPH, SESN2,SLC1A4,SLC1A5,SLC6A9,SLC7A11,SLC7A5,TRIB3,WARS1
	LM	3.277	ABCG8,ATF5,BCAT1,BGLAP, CHAC1,CTH, FGF21,MTHFD2,SESN2,SLC1A4,SLC1A5,SLC7A11,SLC7A5,TRIB3,WARS1
CDKN2A	LH	2.578	AURKB, BCAT1,BIRC5,BUB1B, CCL5,CCNA2,CCNB1,CCR7,CDC25C, CDK1,CTH, GBP1,IL20,LEF1,MKI67,MRC1,MYBL2,PLK1,PSAT1,RORA, RRM2
	LK	4.155	ASF1B, AURKB, BARD1,BCAT1,BIRC5,BLM, BUB1B, CCL5,CCNA2,CCNB1,CCR7,CDC25C, CDK1,CTH, E2F1,E2F2,FBXO5,FST, GBP1,GTSE1,IL20,IL2RG, KIFC1,LEF1,MAD2L1,MCM4,MCM5,MELK, MKI67,MMP1,MYBL2,PLK1,PSAT1,PSMB9,PSPH, RAD51AP1,RECQL4,RORA, RRM2,TGFBI
	LM	2.087	AURKB, BCAT1,BIRC5,CCNA2,CCNB1,CCR7,CTH, GTSE1,IL17B, IL20,LEF1,MKI67,PLK1,RAD51AP1,TGFBI
IRF1	LH	3.963	CCL5,CCNB1,CMPK2,GBP2,IDO1,IFI44,IFI6,IFIH1,IFIT1,IFIT2,IFIT3,IFITM1,ISG15,MX1,OAS1,RIGI, RSAD2,TNFSF10
	LK	4.607	BRIP1,CCL20,CCL5,CCNB1,CMPK2,CXCL8,E2F1,EIF2AK2,GBP2,IDO1,IFI44,IFI44L, IFI6,IFIH1,IFIT1,IFIT2,IFIT3,IFIT5,IFITM1,IFITM3,IRF9,ISG15,MX1,OAS1,PSMB9,RIGI, RSAD2,SOCS1,SP110,TAP1,TMEM140,TNFSF10
	LM	2.965	CCNB1,GBP2,IFIT1,IFIT2,IFIT3,IFITM1,IL27,ISG15,OAS1,RSAD2
IRF3	LH	4.134	CCL5,CMPK2,GBP1,IFI44,IFI6,IFIH1,IFIT1,IFIT2,IFIT3,IL20,ISG15,LCN2,OAS1,RIGI, RSAD2,TNFSF10,USP18,ZBP1
	LK	5.247	CCL5,CMPK2,CXCL8,DHX58,EIF2AK2,FST, GBP1,IFI44,IFI44L, IFI6,IFIH1,IFIT1,IFIT2,IFIT3,IFITM3,IL20,ISG15,ISG20,LCN2,OAS1,PARP14,RIGI, RSAD2,TAP1,TNFSF10,TNFSF15,UBE2L6,USP18,ZBP1
	LM	3.074	IFIT1,IFIT2,IFIT3,IL20,IL27,ISG15,LCN2,OAS1,RSAD2,TNFSF15,ZBP1
IRF5	LH	3.248	CCL5,CMPK2,IFI44,IFIH1,IFIT1,IFIT2,IFIT3,ISG15,OAS1,RIGI, RSAD2,TNFSF10
	LK	3.915	CCL5,CMPK2,DHX58,IFI44,IFIH1,IFIT1,IFIT2,IFIT3,IFITM3,ISG15,ISG20,OAS1,RIGI, RSAD2,SP110,TNFSF10,UBE2L6
	LM	2.584	CXCR4,IFIT1,IFIT2,IFIT3,ISG15,OAS1,RSAD2
IRF7	LH	4.663	CCL5,CMPK2,GBP1,HERC5,IDO1,IFI44,IFI6,IFIH1,IFIT1,IFIT2,IFIT3,IFITM1,ISG15,ITGAX, MX1,MX2,OAS1,RIGI, RSAD2,TNFSF10,UBA7,USP18,ZBP1
	LK	5.68	CCL5,CMPK2,DHX58,GBP1,HERC5,IDO1,IFI44,IFI44L, IFI6,IFIH1,IFIT1,IFIT2,IFIT3,IFITM1,IFITM3,IRF9,ISG15,ISG20,ITGAX, MX1,MX2,OAS1,PARP14,PSMB9,RIGI, RSAD2,RTP4,SOCS1,TAP1,TNFSF10,UBA7,UBE2L6,USP18,ZBP1
	LM	2.924	IFIT1,IFIT2,IFIT3,IFITM1,IL27,ISG15,MAP3K8,OAS1,RSAD2,ZBP1
IRF9	LH	3.252	CMPK2,GBP1,Ifi27,IFIT1,IFIT2,IFIT3,IFITM1,ISG15,MX1,RIGI, RSAD2,TNFSF10,USP18,ZBP1
	LK	3.62	CMPK2,CXCL8,GBP1,HERC6,Ifi27,IFIT1,IFIT2,IFIT3,IFITM1,IFITM3,ISG15,ISG20,MX1,PARP14,RIGI, RSAD2,RTP4,SOCS1,TNFSF10,USP18,ZBP1
	LM	2.416	CCN4,IFIT1,IFIT2,IFIT3,IFITM1,IL27,ISG15,MYH7,RSAD2,ZBP1
KAT5	LH	2.449	ALDH1L2,CACNB2,CHAC1,CTH, SCN2A, TTR, WARS1
	LK	3.207	ALDH1L2,APOA1,CACNB2,CHAC1,CTH, CXCL8,FST, GTPBP2,MAD2L1,PCK2,PLA2G12A, PSPH, SCN2A, SLC6A9,SPAG11B, TTR, WARS1
	LM	2.646	ALDH1L2,CACNB2,CHAC1,CHRM1,CTH, CXCR4,SCN2A, SPAG11B, WARS1
KDM5B	LH	2.354	AURKA, BUB1B, CCNB1,CDC25C, CDCA3,CDK1,DLGAP5,ISG15,PBK, TOP2A
	LK	4.113	AURKA, BRCA1,BUB1B, CCNB1,CDC25C, CDCA3,CDK1,DLGAP5,E2F1,ECT2,FBXO5,FST, HMMR, INSIG1,ISG15,KIF2C, MCM2,MCM3,MCM5,NCAPH, NDC80,PBK, TOP2A, TTK
	LM	2.134	AURKA, CCNB1,CDCA3,DLGAP5,INSIG1,ISG15,MCAM, TOP2A
MEF2A	LH	2.813	GBP2,GSTA3,IFI44,IFIT2,IFIT3,MYOCD, OAS1,RSAD2
	LK	2.132	ACTC1,ASB4,GBP2,IFI44,IFIT2,IFIT3,ISG20,MYOCD, OAS1,RSAD2
	LM	2.373	ASB4,GBP2,GSTA3,IFIT2,IFIT3,IL27,MYOCD, OAS1,RSAD2,STAR
NONO	LH	4.539	CCL5,CMPK2,GBP1,HERC5,IDO1,IFI44,IFI6,IFIH1,IFIT1,IFIT2,IFIT3,IFITM1,ISG15,MX1,MX2,OAS1,RIGI, RSAD2,TNFSF10,TNFSF18,USP18
	LK	5.641	BRIP1,CCL5,CMPK2,EIF2AK2,EPSTI1,GBP1,HERC5,HERC6,IDO1,IFI44,IFI44L, IFI6,IFIH1,IFIT1,IFIT2,IFIT3,IFIT5,IFITM1,ISG15,ISG20,MX1,MX2,OAS1,PARP14,PARP9,PDE4C, PDE8B, RIGI, RNF213,RSAD2,RTP4,SAMD9,SP110,TNFSF10,TNFSF15,TNFSF18,TRANK1,UBE2L6,USP18
	LM	2.739	IFIT1,IFIT2,IFIT3,IFITM1,ISG15,OAS1,PDE2A, PDE8B, RSAD2,TNFSF15,TNFSF18
NUPR1	LH	3.556	ARHGAP11A, ASPM, AURKA, BUB1,BUB1B, CCDC134,CCNA2,CCNB2,CDC25C, CDCA2,CDCA3,CDCA8,CKAP2L, E2F8,ERCC6L, ESPL1,ETV1,GBP2,HJURP, IFIT2,KIF11,KIF20A, KIF23,LCN2,MCM10,MKI67,MX2,PLK1,RAB17,SHCBP1,SPAG5,SPC24,TRIB3
	LK	3.556	ARHGAP11A, ASPM, AURKA, BUB1,BUB1B, CCDC134,CCNA2,CCNB2,CDC25C, CDCA2,CDCA3,CDCA8,CKAP2L, E2F8,ERCC6L, ESPL1,ETV1,GBP2,HJURP, IFIT2,KIF11,KIF20A, KIF23,LCN2,MCM10,MKI67,MX2,PLK1,RAB17,SHCBP1,SPAG5,SPC24,TRIB3
	LM	3.556	ASPM, AURKA, BGLAP, BUB1,CCDC134,CCNA2,CCNB2,CDCA3,CDCA8,CKAP2L, CXCR4,EPGN, ESPL1,FANCD2,GBP2,GTSE1,HJURP, IFIT2,KIF20A, KNL1,LCN2,MAT2A, MKI67,MMP12,PLK1,RAB17,RFTN2,SESN2,SHCBP1,SPAG5,SPC24,SPDL1,TRIB3
PML	LH	2.54	BIRC5,CIT, IFI44,IFIH1,IFIT1,IFIT3,IFITM1,ISG15,MX1,OAS1
	LK	4.122	APOA1,BIRC5,BRCA1,CIT, EPSTI1,FANCD2,HERC6,HMGCS1,IFI44,IFI44L, IFIH1,IFIT1,IFIT3,IFITM1,ISG15,ISG20,MX1,OAS1,PSMB9,STMN1,TAP1
	LM	2.373	BIRC5,CXCR4,FANCD2,HMGCS1,IFIT1,IFIT3,IFITM1,ISG15,OAS1
SPI1	LH	4.18	CCNA2,CCNB1,CCNB2,CCR7,CDK1,CMPK2,IFI44,IFI6,IFIT1,IFIT2,IFIT3,IFITM1,ISG15,LEF1,MRC1,MX1,RSAD2,TNFSF10,USP18
	LK	4.437	CCNA2,CCNB1,CCNB2,CCR7,CDK1,CMPK2,E2F1,E2F2,IFI44,IFI44L, IFI6,IFIT1,IFIT2,IFIT3,IFITM1,IFITM3,IL24,IRF9,ISG15,ISG20,LEF1,MMP1,MX1,OSCAR, PSMB9,RSAD2,SP110,TNFSF10,USP18
	LM	3.274	CCNA2,CCNB1,CCNB2,CCR7,IFIT1,IFIT2,IFIT3,IFITM1,IL24,IL27,ISG15,LEF1,RSAD2,TLX3
STAT1	LH	4.252	BCL2A1,BIRC5,CCL5,CCR7,CMPK2,GBP1,GBP2,IDO1,IFI44,IFI6,IFIH1,IFIT1,IFIT2,IFIT3,IFITM1,ISG15,ITGAX, LCN2,MX1,OAS1,RSAD2,TNFSF10,USP18,WARS1,ZBP1
	LK	5.666	APOA1,BCL2A1,BIRC5,CCL20,CCL5,CCR7,CMPK2,CXCL8,EIF2AK2,EPSTI1,GBP1,GBP2,HERC6,IDO1,IFI44,IFI44L, IFI6,IFIH1,IFIT1,IFIT2,IFIT3,IFITM1,IFITM3,IRF9,ISG15,ITGAX, LCN2,MAFA, MMP1,MX1,OAS1,PARP9,PSMB9,RNF213,RSAD2,RTP4,SLC8A1,SMAGP, SOCS1,SP110,TAP1,TNFSF10,USP18,WARS1,ZBP1
	LM	3.132	BIRC5,CCR7,GBP2,IFIT1,IFIT2,IFIT3,IFITM1,ISG15,LAMA1,LCN2,MYH7,OAS1,PHACTR1,RSAD2,SLC8A1,WARS1,ZBP1
STAT2	LH	2.877	CCL5,GBP1,IFI6,IFIT1,IFIT2,IFIT3,IFITM1,ISG15,MX1,OAS1,RSAD2,TNFSF10,USP18,WARS1,ZBP1
	LK	3.327	CCL5,CXCL8,GBP1,IFI6,IFIT1,IFIT2,IFIT3,IFITM1,IRF9,ISG15,MX1,OAS1,RSAD2,RTP4,SOCS1,TNFSF10,USP18,WARS1,ZBP1
	LM	2.326	GLI1,IFIT1,IFIT2,IFIT3,IFITM1,ISG15,OAS1,RSAD2,WARS1,ZBP1
TCF3	LH	3.108	AURKA, BIRC5,BUB1,CCNA2,CCNB1,CCNB2,CDC20,CDCA3,CDK1,CEP55,E2F8,ESCO2,FGD2,Ifi27,KIF11,KIF4A, LEF1,MKI67,NEK2,NR4A3,RGS16,RIGI, RORA, RRM2,SPC24,ST8SIA1,TOP2A, ZBP1
	LK	3.763	ANLN, ASF1B, AURKA, BIRC5,BUB1,CCNA2,CCNB1,CCNB2,CDC20,CDC45,CDCA3,CDK1,CDKN3,CEP55,CKS2,E2F8,ECT2,ESCO2,FGD2,HMMR, Ifi27,KIF11,KIF2C, KIF4A, LEF1,MAD2L1,MKI67,MXD3,NCAPG, NDC80,NEK2,NR4A3,NUF2,NUSAP1,PLK4,PRC1,RAB33A, RGS16,RIGI, RORA, RRM2,SLC7A5,SMAGP, SOCS1,SPC24,ST8SIA1,TOP2A, TTK, ZBP1
	LM	2.689	AURKA, BIRC5,BUB1,CCNA2,CCNB1,CCNB2,CDC20,CDCA3,CELA1,CEP55,ENTPD1,FGD2,LEF1,MKI67,MYL4,NEK2,NR4A3,NUSAP1,SLC7A5,SPC24,ST8SIA1,TOP2A, ZBP1
TP53	LH	4.159	ABCB1,ARHGAP11A, ASPM, AURKA, AURKB, BCAT1,BCL2A1,BIRC5,BUB1,BUB1B, CARD11,CCL5,CCNA2,CCNB1,CCNB2,CCR7,CDC20,CDC25C, CDC6,CDK1,CENPE, CENPF, CEP55,CTH, CYP19A1,CYP1A1,DLGAP5,DNAJC12,E2F8,ENPP2,ESPL1,FABP3,FAM83D, GBP1,GPR27,HERC5,HJURP, IFI44,IFIH1,IFIT1,IFIT3,INHBA, ISG15,KCNJ2,KIF22,KIF23,LCN2,MKI67,MRC1,MX1,MYBL2,MYOCD, NEK2,NPTX1,NR4A3,NR5A1,OAS1,PBK, PLK1,PTTG1,RGS16,RIGI, RRM2,RSAD2,SLC7A11,TMOD4,TNFSF10,TOP2A, TRIB3,UBA7,UBE2C, WNT4
	LK	6.11	ABCB1,ANLN, APOA1,ARHGAP11A, ARHGAP45,ARHGEF2,ASF1B, ASPM, AURKA, AURKB, BCAT1,BCL2A1,BIRC5,BRCA1,BUB1,BUB1B, CARD11,CCL5,CCNA2,CCNB1,CCNB2,CCR7,CDC20,CDC25C, CDC6,CDK1,CDKN3,CENPA, CENPE, CENPF, CEP55,CHEK2,CKAP2,CKS2,CTH, CXCL8,CYP1A1,DLGAP5,DNAJC12,DUSP4,E2F1,E2F2,E2F8,ESPL1,FABP3,FAM83D, FANCD2,FOXM1,FUT1,GAS1,GBP1,GDF15,GFY, GPR27,GPR62,GTSE1,HERC5,HJURP, HMGCS1,HMMR, IFI44,IFI44L, IFIH1,IFIT1,IFIT3,INSYN2A, IRF9,ISG15,KCNB1,KCNJ2,KIF18A, KIF22,KIF23,KIF24,KIFC1,KNTC1,KRT14,LCN2,MAD2L1,MCM2,MCM3,MCM4,MCM5,MELK, mir-191,MIS18A, MIS18BP1,MKI67,MMP1,MX1,MYBL2,MYOCD, NCAPG, NCAPH, NDC80,NEK2,NPTX1,NR4A3,NR5A1,NUSAP1,OAS1,ORM1,PBK, PCLAF, PLK1,POLE2,PRC1,PSMB9,PTP4A3,PTTG1,RAD51AP1,RECQL4,RGS16,RIGI, RRM2,RSAD2,SERPINB9,SESN2,SGTB, SLC7A11,SLC7A5,SMC2,SPDL1,STMN1,TAP1,TGFBI, TMOD4,TNFSF10,TNFSF15,TOP2A, TP73,TPX2,TRIB3,TRIM6,TTK, UBA7,UBE2C, UBE2L6,UHRF1,UNC5B, UVRAG, VCAN, ZAP70
	LM	2.291	ABCB1,ARHGAP45,ASPM, AURKA, AURKB, BCAT1,BGLAP, BIRC5,BUB1,CCNA2,CCNB1,CCNB2,CCR7,CDC20,CDC6,CENPE, CEP55,CHEK2,CTH, CYP19A1,DLGAP5,DNAJC12,ESPL1,FABP3,FANCD2,FRMD4A, FUT1,GLI1,GPR27,GTSE1,HJURP, HMGCS1,IFIT1,IFIT3,ISG15,KIF22,LCN2,LRRC3,MAP3K8,MCAM, MKI67,MTHFD2,MYH7,MYL4,MYOCD, NEK2,NPTX1,NR4A3,NR5A1,NUSAP1,OAS1,PDE2A, PDGFRA, PLK1,PTP4A3,PTTG1,RAD51AP1,RSAD2,SESN2,SGTB, SLC7A11,SLC7A5,SPDL1,TGFBI, TGM2,TNFSF15,TOP2A, TPX2,TRIB3,WNT4,ZAP70
TP73	LH	2.263	ABCB1,AQP5,AURKA, BIRC5,CCNA2,CCNB1,CCR7,CDC20,CDC25C, CDK1,CDKN1C, CTH, Elob, H2BC11,ITGAX, KIF22,KIF23,TAS1R1,TTR, UBE2C, WNT4
	LK	3.175	ABCB1,AURKA, BIRC5,CCNA2,CCNB1,CCR7,CDC20,CDC25C, CDH22,CDK1,CDKN1C, CDKN3,CKS2,CTH, E2F1,FST, GTSE1,H2BC11,ITGAX, KIF22,KIF23,MTX3,MYOZ2,PROCR, PTP4A3,SLC4A3,STMN1,TAP1,TAS1R1,TP73,TPX2,TTR, UBE2C, UVRAG
	LM	2.751	ABCB1,AURKA, BIRC5,CCNA2,CCNB1,CCR7,CDC20,CTH, DMC1,Elob, GTSE1,H2BC11,KIF22,PROCR, PTP4A3,STARD8,TPX2,WNT4
TRPS1	LH	3.317	AURKB, BUB1,CCNA2,CCNB2,CDC20,CDC25C, CDK1,CENPF, KIF11,PTTG1,TOP2A
	LK	3.606	AURKB, BUB1,CCNA2,CCNB2,CDC20,CDC25C, CDK1,CENPF, FOXM1,KIF11,PTTG1,TOP2A, TPX2
	LM	2.985	AURKB, BGLAP, BUB1,CCNA2,CCNB2,CDC20,PTTG1,TOP2A, TPX2
ZBTB10	LH	3.843	BATF3,BIRC5,CCL5,CCR7,IDO1,IFIH1,IFIT1,IFIT2,IFIT3,IL20,ISG15,NR4A3,OAS1,PRKCQ, RIGI, ZBP1
	LK	4.768	BATF3,BIRC5,CCL5,CCR7,DHX58,DTX3L, IDO1,IFIH1,IFIT1,IFIT2,IFIT3,IFITM3,IL20,ISG15,ISG20,NR4A3,OAS1,PARP9,PRKCQ, RIGI, RTP4,TNFSF15,TRIM6-TRIM34,ZBP1
	LM	3.286	BIRC5,CCR7,IFIT1,IFIT2,IFIT3,IL20,IL27,ISG15,NR4A3,OAS1,TNFSF15,ZBP1

**Table 2:** Negatively regulated upstream transcription regulators in bovine mammary epithelial cells 60 h after histidine (LH), lysine (LK) or methionine (LM) subtraction to ¼ the normal concentration

Upstream Regulator	Treatments	Activation z-score	Target Molecules in Dataset
CCND1	LH	-2.887	ASPM, AURKA, BIRC5,CCNA2,CDC6,CDCA2,CDCA8,CENPF, CEP55,COL20A1,E2F8,ENPP2,ESCO2,FAM83D, GAS2L3,HJURP, KIF11,KIF20A, KIF4A, MCM10,MYRIP, PLK1,RRM2
	LK	-3.955	ARHGEF2,ASPM, AURKA, BIRC5,BRCA1,BRIP1,CCNA2,CDC45,CDC6,CDCA2,CDCA7,CDCA8,CENPF, CEP55,COL20A1,DEPDC1,DTL, E2F1,E2F8,ESCO2,FAM83D, FOXM1,GAS2L3,HJURP, KIF11,KIF20A, KIF20B, KIF2C, KIF4A, KNL1,KRT14,MCM10,MCM4,MELK, MTFR2,MYRIP, NCAPH, PCLAF, PLK1,PSMC3IP, RRM2,TPX2,TRIP13,UHRF1
	LM	-2.828	ASPM, AURKA, BIRC5,CCNA2,CDC6,CDCA7,CDCA8,CEP55,EPGN, GAS2L3,HJURP, KIF20A, KIF20B, KNL1,MYRIP, PLK1,TPX2
CITED2	LH	-2.704	CMPK2,GBP2,IFI44,IFIH1,IFIT2,IFIT3,ISG15,MRC1,NR5A1,OAS1,RIGI, RSAD2,WARS1,ZBP1
	LK	-4.613	CMPK2,CXCL8,DTX3L, GBP2,IFI44,IFIH1,IFIT2,IFIT3,IRF9,ISG15,MMP1,NR5A1,OAS1,PARP14,PARP9,RIGI, RSAD2,RTP4,SLC1A3,TMEM140,TNFSF15,TRIM6-TRIM34,TTC39B, WARS1,ZBP1
	LM	-2.666	ENTPD1,GBP2,IFIT2,IFIT3,ISG15,NR5A1,OAS1,RSAD2,TGM2,TNFSF15,TTC39B, WARS1,ZBP1
E2F3	LH	-2.724	BIRC5,CCNA2,CCNB1,CCNB2,CDC6,CDCA3,CDK1,H2BC11,H2BC15,INHBA, MCM10,MYBL2,PLK1,PTTG1,RRM2,UBE2C
	LK	-3.97	AKR1C3,BIRC5,CCL20,CCNA2,CCNB1,CCNB2,CDC45,CDC6,CDCA3,CDK1,E2F1,E2F2,FBXO5,FST, H2BC11,MAD2L1,MCM10,MCM2,MCM3,MCM4,MCM5,MYBL2,NCAPG2,ORC1,PCLAF, PLK1,PTTG1,RRM2,TP73,TPX2,UBE2C
	LM	-2.158	BIRC5,CCNA2,CCNB1,CCNB2,CDC6,CDCA3,H2BC11,PLK1,PTTG1,TPX2
ETV3	LH	-3.873	GBP1,HERC5,IFI44,IFI6,IFIH1,IFIT1,IFIT2,IFIT3,LGALS9,MX1,MX2,OAS1,RIGI, RSAD2,USP18
	LK	-5.186	DHX58,DTX3L, EIF2AK2,GBP1,GTPBP2,HERC5,IFI44,IFI44L, IFI6,IFIH1,IFIT1,IFIT2,IFIT3,IFIT5,IRF9,LGALS9,MMP1,MX1,MX2,OAS1,PARP9,PSMB9,RIGI, RSAD2,SAMD9,SP110,USP18
	LM	-2.236	IFIT1,IFIT2,IFIT3,OAS1,RSAD2
ETV6	LH	-3.422	CMPK2,DLL4,IFI44,IFIT1,IFIT3,IFITM1,ISG15,MX1,MX2,OAS1,RIGI, RSAD2
	LK	-4.967	CMPK2,DHX58,DLL4,DTX3L, EIF2AK2,IFI44,IFI44L, IFIT1,IFIT3,IFIT5,IFITM1,ISG15,MX1,MX2,OAS1,PARP14,PARP9,RIGI, RSAD2,SAMD9,SP110,SPRY4,TRANK1,UBE2L6,ZNFX1
	LM	-2.598	DLL4,IFIT1,IFIT3,IFITM1,ISG15,OAS1,RSAD2
FOXM1	LH	-4.182	ASPM, AURKA, AURKB, BIRC5,BUB1B, CCL5,CCNA2,CCNB1,CCNB2,CDC20,CDC25C, CDCA2,CDCA3,CDCA8,CDK1,CENPE, CENPF, HSD11B2,KIF20A, KIF23,LEF1,MKI67,MYOCD, NEK2,PLK1,PTTG1,RRM2,TOP2A
	LK	-4.797	ASPM, AURKA, AURKB, BIRC5,BRIP1,BUB1B, CCL5,CCNA2,CCNB1,CCNB2,CDC20,CDC25C, CDCA2,CDCA3,CDCA8,CDK1,CDKN3,CENPA, CENPE, CENPF, CKS2,FOXM1,GTSE1,HSD11B2,KIF20A, KIF23,KIF2C, LEF1,MKI67,MYOCD, NEK2,NUF2,PLK1,PLK4,PRC1,PTTG1,RRM2,STMN1,TOP2A, VCAN
	LM	-3.427	ASPM, AURKA, AURKB, BIRC5,CCNA2,CCNB1,CCNB2,CDC20,CDCA3,CDCA8,CENPE, GTSE1,HSD11B2,KIF20A, LEF1,MKI67,MYOCD, NEK2,PLK1,PTTG1,TOP2A
FOXO1	LH	-2.785	ASPM, BCL2A1,BIRC5,CCNA2,CCNB1,CCNB2,CCR7,CDK1,CDKN1C, CENPF, DLGAP5,FGF18,FGF21,KIF11,NEK2,NRP2,PBK, TNFSF10,TRIB3,WNT4
	LK	-2.158	AFF3,ANLN, ASPM, BCL2A1,BIRC5,BRIP1,CCL20,CCNA2,CCNB1,CCNB2,CCR7,CDK1,CDKN1C, CENPF, CXCL8,DEPDC1,DLGAP5,EDNRB, FGF18,FGF21,FOXM1,FST, GFPT2,KIF11,KIF18A, MAFA, MCM5,MMP1,NCAPG, NEK2,NRP2,NUSAP1,PBK, PCK2,PRC1,TNFSF10,TRIB3,TXNIP
	LM	-2.254	ASPM, BGLAP, BIRC5,CCNA2,CCNB1,CCNB2,CCR7,CIDEA, COMP, DLGAP5,FGF18,FGF21,FGF23,NEK2,NUSAP1,SEMA6D, TIE1,TRIB3,WNT4
MITF	LH	-3.606	AURKB, CCNB1,CDCA3,CDCA8,CENPF, CEP55,ESPL1,KIF20A, KIF4A, PLK1,SPAG5,SPC24,UBE2C
	LK	-3.927	AURKB, BRCA1,CCNB1,CDCA3,CDCA8,CENPF, CENPM, CEP55,CHTF18,CXCL8,ECT2,EDNRB, ESPL1,KIF20A, KIF4A, KIFC1,MCM2,MCM4,MCM5,NUF2,OSCAR, PLK1,POLE2,PSMC3IP, RECQL4,SPAG5,SPC24,TACC3,TPX2,TXNIP, UBE2C, UVRAG, ZNF114
	LM	-3.742	AURKB, CCNB1,CDCA3,CDCA8,CENPM, CEP55,CHTF18,ESPL1,GLI1,KIF20A, MYL4,PHACTR1,PLK1,SPAG5,SPC24,TPX2
MYB	LH	-2.174	AURKA, BIRC5,CCNB1,CDK1,LCN2,SLC1A5
	LK	-2.574	AURKA, BIRC5,BRCA1,CCNB1,CDK1,FOXM1,LCN2,MMP1,SLC1A5
	LM	-2.207	AURKA, BIRC5,CCNB1,CXCR4,LCN2,MAT2A, PDE2A, SLC1A5,SLC2A3
MYBL2	LH	-3.521	AURKA, BIRC5,BUB1,CCNA2,CCNB1,CCNB2,CDCA3,CDK1,CENPE, MYBL2,PLK1,RRM2,SLC1A5,TOP2A, UBE2C
	LK	-3.655	AURKA, BIRC5,BUB1,CCNA2,CCNB1,CCNB2,CDCA3,CDK1,CENPE, FOXM1,MYBL2,PLK1,RRM2,SLC1A5,TOP2A, UBE2C
	LM	-2.929	AURKA, BIRC5,BUB1,CCNA2,CCNB1,CCNB2,CDCA3,CENPE, PLK1,SLC1A5,TOP2A
MYC	LH	-3.536	ASCL2,AURKB, BCAT1,BCL2A1,BIRC5,BUB1,BUB1B, CCL5,CCNA2,CCNB1,CCNB2,CDC20,CDC25C, CDK1,DLGAP5,F2,GBP2,HERC5,HSD11B2,IFI44,IFI6,IFIH1,IFIT1,IFIT2,IFIT3,INHBA, ISG15,ITGAX, MKI67,MRC1,MX1,MX2,NEK2,OAS1,PLK1,PSAT1,REN, RRM2,RSAD2,SLC1A5,TNFSF10,UBE2C, USP18
	LK	-3.944	ASCL2,AURKB, BCAT1,BCL2A1,BIRC5,BRCA1,BUB1,BUB1B, CCL5,CCNA2,CCNB1,CCNB2,CDC20,CDC25C, CDCA7,CDK1,CDKN3,CKS2,CXCL8,DLGAP5,DUSP4,E2F1,E2F2,F2,FOXM1,GAS1,GBP2,HERC5,HSD11B2,IFI44,IFI44L, IFI6,IFIH1,IFIT1,IFIT2,IFIT3,IFIT5,IRF9,ISG15,ISG20,ITGAX, KIF2C, KRT14,KRT27,MAD2L1,MCM2,MCM5,MKI67,MTFR2,MX1,MX2,NECAB3,NEK2,OAS1,PHYHIP, PLK1,PSAT1,PTP4A3,REN, RPS29,RRM2,RSAD2,SLC1A5,SLC7A5,ST3GAL1,STMN1,TNFSF10,TP73,TPX2,TXNIP, UBE2C, USP18
	LM	-2.907	AURKB, BCAT1,BIRC5,BUB1,CCN4,CCNA2,CCNB1,CCNB2,CDC20,CDCA7,CXCR4,DLGAP5,F2,GBP2,GPT, HSD11B2,IFIT1,IFIT2,IFIT3,ISG15,MAT2A, MKI67,MYH7,NEK2,OAS1,PDGFRA, PLK1,PTP4A3,REN, RPS15A, RSAD2,SLC1A5,SLC2A3,SLC7A5,TPX2,TRIM71
MYOD1	LH	-4.319	BUB1B, CCNB2,CDC20,CDC6,CDCA3,CDK1,CDKN1C, CENPE, CENPF, CEP55,DLGAP5,ENPP2,FAM83D, HJURP, IFIT2,KIF14,KIF4A, MKI67,MX2,NPTX1,PBK, PLK1,RRM2,SHCBP1,SPC24,TOP2A, TROAP, UBA7
	LK	-5.249	ACTC1,ADCY7,BRCA1,BUB1B, CCNB2,CDC20,CDC6,CDCA3,CDK1,CDKN1C, CENPE, CENPF, CEP55,DEPDC1,DLGAP5,E2F2,EIF2AK2,FAM83D, FOXM1,GINS2,HJURP, IFIT2,KIF14,KIF18B, KIF2C, KIF4A, KNL1,LMNB2,MKI67,MMP1,MX2,NCAPG, NCAPH, NDC80,NPTX1,NUSAP1,PBK, PCLAF, PLK1,RRM2,SHCBP1,SPC24,TOP2A, TPX2,TRIP13,TROAP, UBA7
	LM	-3.494	ADCY7,CCNB2,CDC20,CDC6,CDCA3,CENPE, CEP55,DLGAP5,HJURP, IFIT2,KNL1,MKI67,MYH7,MYL4,MYMK, NPTX1,NUSAP1,PLK1,SHCBP1,SPC24,TOP2A, TPX2,TROAP
TBX2	LH	-3.302	AURKA, AURKB, BUB1,CCNA2,CCNB1,CDC6,CDCA3,CDK1,E2F8,PLK1,TRIB3
	LK	-4.99	ANLN, ASF1B, AURKA, AURKB, BUB1,CCNA2,CCNB1,CDC6,CDCA3,CDK1,CKAP2,E2F1,E2F8,FOXM1,MAD2L1,MCM2,MCM4,MCM5,MXD3,NCAPG2,PLK1,PRC1,SGO1,SMC2,TRIB3
	LM	-3.162	ATF5,AURKA, AURKB, BUB1,CCNA2,CCNB1,CDC6,CDCA3,PLK1,TRIB3
TOX	LH	-2.303	BUB1,BUB1B, CCNA2,CCNB1,CCR7,CDC20,CDC25C, CDKN1C, ESPL1,LEF1,PLK1
	LK	-3.283	BLM, BRIP1,BUB1,BUB1B, CCNA2,CCNB1,CCR7,CDC20,CDC25C, CDKN1C, ESPL1,LEF1,MAD2L1,MCM5,PLK1,RAD54L, TTK
	LM	-3	BUB1,CCNA2,CCNB1,CCR7,CDC20,ENTPD1,ESPL1,LEF1,PLK1

### Single EAA deficiencies decrease DNA and protein syntheses in BMEC

**Fig. 4:**
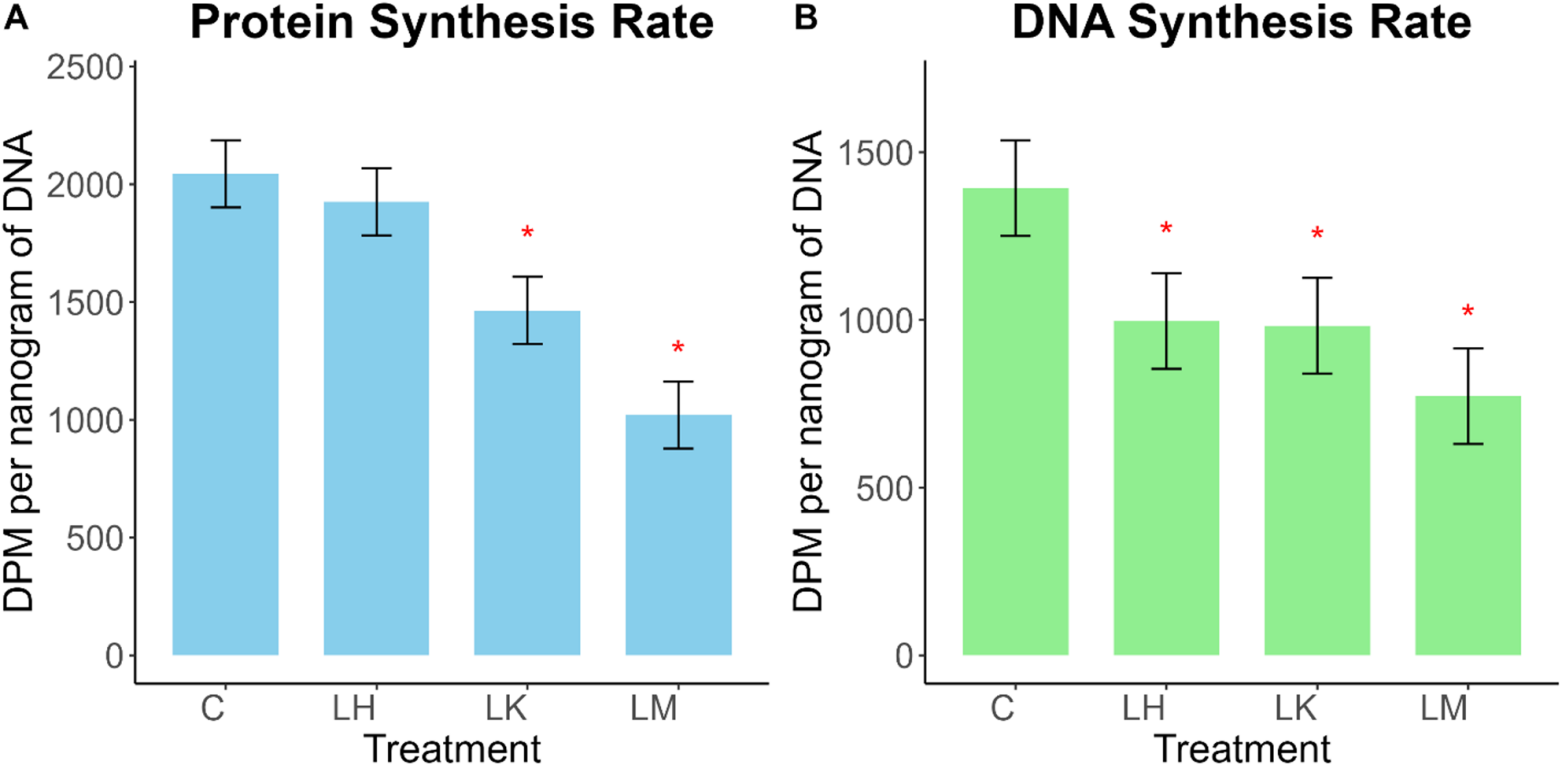
(**A**) Protein and (**B**) DNA synthesis rates in bovine mammary epithelial cells 48 h after histidine (LH), lysine (LK), or methionine (LM) subtraction to ¼ the normal concentration. Plotted values are least square means ± SE (*n* = 3); * indicates *P* < 0.05 compared to CTL

The LH treatment did not affect (*P* = 0.58) protein synthesis rates, whereas LK (*P* = 0.03) and LM (*P* < 0.01) significantly reduced protein synthesis rates (Fig. 4A). Additionally, DNA synthesis rates were decreased by LH (*P* = 0.01), LK (*P* = 0.01), and LM (*P* < 0.01) (Fig. 4B).

## Discussion

EAA deficiency affects protein synthesis acutely in mammary epithelial cells through mTORC1 and eIF2 signalling [3, 4]. Evidence is accumulating that the long-term response to a chronic EAA deficiency involves a change in secretory epithelial mass driven by modifications in protein synthesis, cell proliferation and differentiation [5]. After 48 h of exposing differentiated BMEC to media containing ¼ the normal concentration of histidine, lysine, or methionine, holding all other EAA at normal physiological concentrations [13, 14], we show that fractional DNA synthesis rate was reduced 30 to 40%. Protein synthesis rate per cell, expressed relative to DNA mass, was not affected by histidine deficiency but was reduced 30 to 50% by lysine and methionine deficiencies. To identify potentially novel TF mediating the effects of single EAA deficiency on BMEC activity, we conducted a genome-wide survey of gene expression profiles. Transcriptomes of BMEC exposed to 60 h of histidine, lysine or methionine deficiencies were subjected to RNA-Seq analysis, and upstream transcriptional regulators of differentially expressed genes were given a z-score by IPA software for how likely they were activated or inhibited according to the number of their targets that changed in the direction expected from the literature database.

### EAA deficiencies decrease BMEC proliferation

The RNA-Seq analysis segregated CTL, LH, LK, and LM treatments according to their gene expression signatures as shown by PCA. The top differentially expressed pathways in all three EAA deficiency treatments included kinetochore metaphase signaling, mitotic roles of polo-like kinase, and G2/M DNA damage checkpoint regulation, all of which are related to a smaller number of cells in the cultures progressing through mitosis. This finding is consistent with the lower rates of DNA synthesis measured by [^3^H]thymidine incorporation.

Kinetochore metaphase signaling, which was the most strongly affected pathway in all three treatments, refers to the spindle assembly checkpoint initiated by improperly attached kinetochores during metaphase. The kinetochore is a protein complex assembled on the centromere of chromosomes during cell division, serving as the attachment point for spindle microtubules to ensure accurate segregation of chromosomes into daughter cells. Targets of the checkpoint pathway [20] that were down-regulated by EAA deficiency include cell division cycle 20 (CDC20), aurora kinase B (AURKB), BUB1 mitotic checkpoint serine/threonine kinase (BUB1), and BUB1B. Polo-like kinase 1 (PLK1), whose expression was affected negatively by EAA deficiency, localizes at the kinetochore and is involved in the spindle assembly checkpoint, but PLK1 also has wider roles in mitosis. It is involved in centrosome maturation, along with AURKA [21] which was also down-regulated by EAA deficiency. Decreased expression of PLK1, cyclin B1 (CCNB1), cyclin-dependent kinase 1 (CDK1) and CDC25C is characteristic of the G2/M DNA damage checkpoint that prevents cells from entering mitosis [22]. PLK1 is also involved in the segregation of chromosomes and cytoplasm to daughter cells [23].

Our DEG and isotope results show that a histidine, methionine or lysine deficiency negatively affects BMEC proliferation and DNA synthesis. Previous research also supports a negative effect of EAA deprivation or a positive effect of EAA supplementation on progression through the cell cycle in various cell types [24, 25]. The effect of EAA on cell number via control of proliferation may be an important contributing factor to EAA effects on milk protein yields from the mammary glands of lactating cows. The yield of milk components such as protein and fat depend on the total number of epithelial secretory cells in the mammary glands and their synthetic activity. Indeed, changes in mammary epithelial mass have been implicated in the effects of dam nutrition on milk component yields [5, 26, 27] and justify investigations into the mechanisms by which secretory cell number is modified.

### Interferon signaling is stimulated by EAA deficiency

Several interferon-related genes were significantly increased by EAA deficiency treatments, including interferon-induced protein with tetratricopeptide repeats 1 (IFIT1) and IFIT3 across all treatments, interferon regulatory factor 9 (IRF9) in LH, IRF1, IRF3, IRF7, IRF9 in LK, and IRF6 in LM. Activation z-scores assigned by IPA software highlight a network of interferon-related regulators and processes predicted to be up-regulated by each of the EAA deficiencies, including all seven members of the family of signal transducers and activators of transcription STAT1, STAT2, STAT3, STAT4, STAT5A, STAT5B, and STAT6. The involvement of the STAT family of TF in mammary epithelial function was first encountered as the mechanism by which prolactin promotes the expression of genes encoding milk proteins [28]. Similarities to the Janus kinase-STAT pathway that had just been revealed in the study of mechanisms of interferon action [29] led to confirmation that dimers of STAT5A/B bound to gamma interferon activation sites on the promoters of milk protein genes in response to prolactin stimulation [30]. Since then, STAT family siblings STAT6 and STAT3 have been implicated in mammary alveologenesis and involution [31].

Interferon signaling is normally thought of as an antimicrobial response of the innate immune system. The presence of foreign double-stranded DNA in the cytosol of immune cells activates the ER-resident stimulator of interferon response cGAMP interactor 1 (STING1) to produce a type I interferon response involving up-regulation of IRF and IFIT expression [32]. Epithelial cells of the mammary glands are also capable of mounting this interferon response when exposed to infectious microbes [33]. IRF proteins are tumor suppressors that induce cell cycle arrest and apoptosis in many types of cancer cells, including breast cancer [34, 35]. Abundance of IRF1 is approximately 30% lower in human breast cancer compared to normal breast tissue [36]. In the normal mammary epithelial cell, IRFs also promote cell cycle arrest and p53-independent apoptosis [37–39]. Furthermore, they suppress branching morphogenesis and ribosome biogenesis [40, 41], and contribute to mammary involution upon cessation of milk removal [42, 43]. Interferon-induced IFIT proteins exert antiviral actions by competing with eIF4E to bind to the nonmethylated 5′-cap of viral mRNA and inhibit their translation [44, 45]. Interferons also inhibit the translation of cellular mRNA by activating the eIF2α kinase PKR which is considered part of the integrated stress response and results in greater ATF4 translation [46].

Expression of IRF genes is modulated as epithelial cells progress through the various stages of mammary development [37, 43, 47, 48] but their activation by EAA deprivation has not been shown previously in mammary cells, to our knowledge. Studies of ATF4 knockdown and overexpression indicate that the interferon-stimulated IRF and IFIT genes are downstream targets of ATF4 in a range of normal and cancer cells [49–52]. There is also the possibility that physiological DNA damage due to hyperproliferation or apoptosis activates interferon signalling through STING1 [53, 54]. Our results have revealed that interferon signaling via IRF, IFIT, and STAT factors is activated in BMEC by EAA deficiency and may contribute to the observed reduced rates of cell proliferation and protein synthesis. Interferon signaling is a novel candidate for mediating the effects of EAA supply on milk protein yield in lactating cows.

### Key upstream transcriptional regulators of EAA deficiency effects on BMEC proliferation and apoptosis

The transcriptional effects of EAA deficiency are classically understood to be mediated by ATF4 following phosphorylation of eIF2α by GCN2 in the free tRNA-activated arm of the integrated stress response [46]. It has been confirmed by knockout of the gene for GCN2 that EAA deficiency causes eIF2α phosphorylation in BMEC through this particular eIF2 kinase [55]. The phosphorylation of eIF2α serves as a regulatory nexus, tempering general protein synthesis as an inhibitor of eIF2 activation while selectively enhancing the translation of ATF4. ATF4 homo- and hetero-dimerizes with ATF6, FOS, and JUN, among other basic leucine-zipper transcription factors, to induce expression of genes involved in resolving and adapting to the EAA deficiency, such as amino acid transporters and ER-resident proteins, in what has been called the amino acid response pathway [7]. We previously found that ATF4 expression increases in BMEC in response to LK and LM [6], and Edick et al. [55] showed that LK or the combined deprivation of arginine, leucine, and lysine induced the expression of ATF4 downstream targets ASNS and solute carrier family 7 member 1 (SLC7A1). Here, we extend these findings to show an array of classic ATF4 targets increasing in BMEC in response to deficiencies of histidine, lysine or methionine, such as amino acid transporters (SLC1A4, SLC1A5, SLC7A5, SLC7A11), the branched-chain aminotransferase BCAT1, two aminoacyl-tRNA synthetases (GARS1, WARS1), the sestrin2 leucine sensor SESN2, and regulators of apoptosis (CHAC1, TRIB3). ATF4 can enhance the mTORC1 pathway by increasing the availability of AAs in cells through the AA transporters SLC7A1 and SLC7A5, which subsequently promotes protein synthesis [56–58]. Conversely, ATF4 can also suppress the mTORC1 pathway via SESN2 and DDIT4, inhibiting protein synthesis [9, 58–60]. Moreover, ATF4 induces cell apoptosis through the CHOP/ATF4 pathway, targeting downstream genes CHAC1 and TRIB3 to cause cell death [61, 62], which could further inhibit milk protein yield. Those findings and our results show that ATF4 is a key TF in the response to EAA deficiency.

An alternative to the ATF4 pathway for TF activation by EAA deficiency involves RAS/RAF/MEK/ERK signaling, which is one of the four mitogen-activated protein kinase (MAPK) cascades that stimulate the expression of early-response genes FOS, JUN, and EGR1 [8, 10, 63, 64]. We previously did not find any increases in expression of FOS, JUN, or EGR1 in response to LK or LM [6] and concluded that a prolonged EAA deficiency in BMEC did not invoke the MAPK cascade. The current results support that conclusion, as these TF were not singled out by RNA-Seq data analysis.

The TF MYC can also be upregulated by the RAS/RAF/MEK/ERK pathway [65–67] and its expression was also not affected by LK or LM in our previous study [6].We did find, however, that hypoxia-inducible factor 1α (HIF1A) increased in expression [6], and HIF1A is thought to be anti-proliferative in large part by displacing the repressor MYC from the promoter of cyclin-dependent kinase inhibitor (CDKN) genes [68]. HIF1A was one of the differentially up-regulated genes in the current data set, and pathway analysis identified MYC as a significantly inhibited TF and CDKN2A as significantly activated. Thus, HIF1A may be a previously unrecognized mediator of EAA deficiency effects.

Other differentially affected TFs related to cell cycle progression commonly shared by the EAA deficiency treatments include FOXM1, CCND1, NUPR1, TP53, and DDIT3. FOXM1 is a transcriptional activator of cell cycle progression, and its down-regulation affects the expression of several cell cycle genes, including CCND1, whose gene product is a regulatory subunit of cyclin-dependent kinases that drives progression through the G1 phase of cell growth and entry into DNA synthesis, and CCNB1 and PLK1 that participate in the G2/M transition [69]. FOXM1 down-regulation may be one of the prime reasons for slower proliferation during EAA deficiency in BMEC. However, whether it is inhibited by ATF4, interferon signaling, or some other pathway remains to be determined. ATF4 silencing increased FOXM1 expression in one study [51] but did not affect FOXM1 in several others [50, 70, 71]. Treatment of glioma cells with a mixture of interferons for 72 h decreased FOXM1 expression, and the authors proposed a mechanism in which TP53 activation by the interferon-induced eIF2α kinase PKR suppresses PLK1 expression, which down-regulates FOXM1 and delays progression into and through mitosis [72].

TP53 is a well-established tumor suppressing TF that induces cell cycle arrest and apoptosis [73]. DDIT3, another TF, is associated with the ER stress response and plays a crucial role in ER stress-induced apoptosis [7]. Both TP53 and DDIT3 interact with ATF4 in various regulatory contexts. TP53 and ATF4 independently converge to regulate numerous common metabolic and pro-apoptotic target genes [74], including SESN2 [75]. Additionally, DDIT3 forms a heterodimer with ATF4, together facilitating apoptosis in cells [76]. Our findings suggest these genes promote apoptosis, potentially through mechanisms involving ATF4.

The stress-responsive TF NUPR1 is hard to categorize as promoting cell proliferation or inducing apoptosis. It was discovered as a stress response gene associated with cell-cycle progression that can aid cells in bypassing the G0/G1 checkpoint and thereby accelerate proliferation [77]. NUPR1 interacts with p53 to promote the progression of mammary epithelial cells through the cell cycle [78, 79], and the knockdown of NUPR1 expression increases apoptosis [80, 81], but some researchers have pointed out that NUPR1 might also stimulate apoptosis. NUPR1 inhibited cell growth and repopulation in human pancreatic cancer cells [82] and breast cancer cells [83]. Specifically, NUPR1 induced apoptosis by upregulating the ER stress-related genes, including TRB3, ATF4, and CHOP (DDIT3) in brain tumor cells [84]. NUPR1 is a downstream target of ATF4 [85] and can be activated by EAA deficiencies, such as leucine deprivation, through ATF4 regulation [86]. It may also play a critical role in ATF4-mediated apoptosis. These results suggest that the effect of NUPR1 on the cell cycle highly depends on the specific cell or tissue. In our BMEC, NUPR1 was activated by EAA deficiencies and cell proliferation was inhibited, which aligns with findings in mammary cancer research [83].

Besides the TF discussed above, several other TFs identified by the upstream regulator analysis could contribute to a slower cell cycle progression, including TP73 and FOXO1. Our results strongly indicate that EAA deficiency inhibits cell proliferation and promotes apoptosis, which are the main determinants of milk secretory cell number. Because daily milk protein yields are highly dependent on the functional secretory cell number within the mammary glands, deficiencies of EAA are likely to decrease milk protein yields not only by limiting the supply of substrate for protein synthesis, but also through decreasing secretory cell number. A limitation of this study is the absence of protein-level or functional validation of the predicted upstream transcriptional regulators. Future studies will be required to verify the functional roles and signaling pathways of these transcription factors.

## Conclusion

This study comprehensively analyzes the transcriptional responses in BMEC under EAA deficiency. Through RNA-sequencing and pathway analysis, we identified key transcription factors and signaling pathways that mediate the effects of EAA deprivation on mammary function. Our results highlight the pivotal role of ATF4, which is significantly activated under histidine, lysine and methionine deprivation. Interferon signaling emerged as a novel pathway activated by EAA deficiency that might contribute to reduced cell proliferation and protein synthesis. The activation of ATF4, the novel role of interferon signaling, and the involvement of HIF1A, FOXM1, TP53, and DDIT3 provide new insights into mechanisms of regulation of milk protein synthesis. These findings enhance our understanding of how nutrient availability impacts mammary gland function, potentially informing strategies to optimize milk production in lactating dairy cows.

## Supplementary Information

Supplementary Material 1.

Supplementary Material 2.

## Data Availability

The datasets generated and analysed during the current study are available in the GEO – Gene Expression Omnibus repository, https://www.ncbi.nlm.nih.gov/geo/query/acc.cgi?acc=GSE269148. The data is currently private, and the review token is yzmlgoqejzglrsl. The data will be published on December 31, 2025.
