# Supplementary material for: An RNA-sequencing analysis to determine potential upstream transcriptional regulators of essential amino acid deficiency responses in bovine mammary epithelial cells
Source: BMC Genomics. 2026 Mar 27;27:439. doi: 10.1186/s12864-026-12791-y (PMC13147841; doi:10.1186/s12864-026-12791-y)
Supplement: Supplementary file 1 — Supplementary Material 1. [file 12864_2026_12791_MOESM1_ESM.docx]

Supplementary Table S1. RNA-sequencing quality control metrics for retained samples

| ***Sample Name*** | ***% Aligned*** | ***% > Q30*** | ***M Seqs*** |
| --- | --- | --- | --- |
| **C1_S398_L002_001_fastq** | 83.6% | 94.0% |  |
| **C1_S398_L002_001_fastq_forward** |  |  | 169.5 |
| **C1_S398_L002_001_fastq_reverse** |  |  | 169.5 |
| **C2_S399_L002_001_fastq** | 88.9% | 93.9% |  |
| **C2_S399_L002_001_fastq_forward** |  |  | 161.4 |
| **C2_S399_L002_001_fastq_reverse** |  |  | 161.4 |
| **C3_S400_L002_001_fastq** |  | 94.2% |  |
| **C3_S400_L002_001_fastq_forward** |  |  | 1.5 |
| **C3_S400_L002_001_fastq_reverse** |  |  | 1.5 |
| **LH1_S401_L002_001_fastq** | 91.7% | 94.3% |  |
| **LH1_S401_L002_001_fastq_forward** |  |  | 127.2 |
| **LH1_S401_L002_001_fastq_reverse** |  |  | 127.2 |
| **LH2_S402_L002_001_fastq** | 91.5% | 93.8% |  |
| **LH2_S402_L002_001_fastq_forward** |  |  | 151.2 |
| **LH2_S402_L002_001_fastq_reverse** |  |  | 151.2 |
| **LH3_S403_L002_001_fastq** | 91.0% | 93.9% |  |
| **LH3_S403_L002_001_fastq_forward** |  |  | 178.7 |
| **LH3_S403_L002_001_fastq_reverse** |  |  | 178.7 |
| **LM1_S404_L002_001_fastq** | 89.1% | 94.0% |  |
| **LM1_S404_L002_001_fastq_forward** |  |  | 170.3 |
| **LM1_S404_L002_001_fastq_reverse** |  |  | 170.3 |
| **LM2_S405_L002_001_fastq** | 86.1% | 93.5% |  |
| **LM2_S405_L002_001_fastq_forward** |  |  | 118.9 |
| **LM2_S405_L002_001_fastq_reverse** |  |  | 118.9 |
| **LM3_S406_L002_001_fastq** | 89.0% | 93.5% |  |
| **LM3_S406_L002_001_fastq_forward** |  |  | 150.0 |
| **LM3_S406_L002_001_fastq_reverse** |  |  | 150.0 |
| **LY1_S407_L002_001_fastq** | 92.4% | 94.0% |  |
| **LY1_S407_L002_001_fastq_forward** |  |  | 162.5 |
| **LY1_S407_L002_001_fastq_reverse** |  |  | 162.5 |
| **LY2_S408_L002_001_fastq** | 91.8% | 94.0% |  |
| **LY2_S408_L002_001_fastq_forward** |  |  | 160.3 |
| **LY2_S408_L002_001_fastq_reverse** |  |  | 160.3 |
| **LY3_S409_L002_001_fastq** | 93.2% | 93.9% |  |
| **LY3_S409_L002_001_fastq_forward** |  |  | 158.6 |
| **LY3_S409_L002_001_fastq_reverse** |  |  | 158.6 |
