# Supplementary figures and images for: An RNA-sequencing analysis to determine potential upstream transcriptional regulators of essential amino acid deficiency responses in bovine mammary epithelial cells
Source: BMC Genomics. 2026 Mar 27;27:439. doi: 10.1186/s12864-026-12791-y (PMC13147841; doi:10.1186/s12864-026-12791-y)

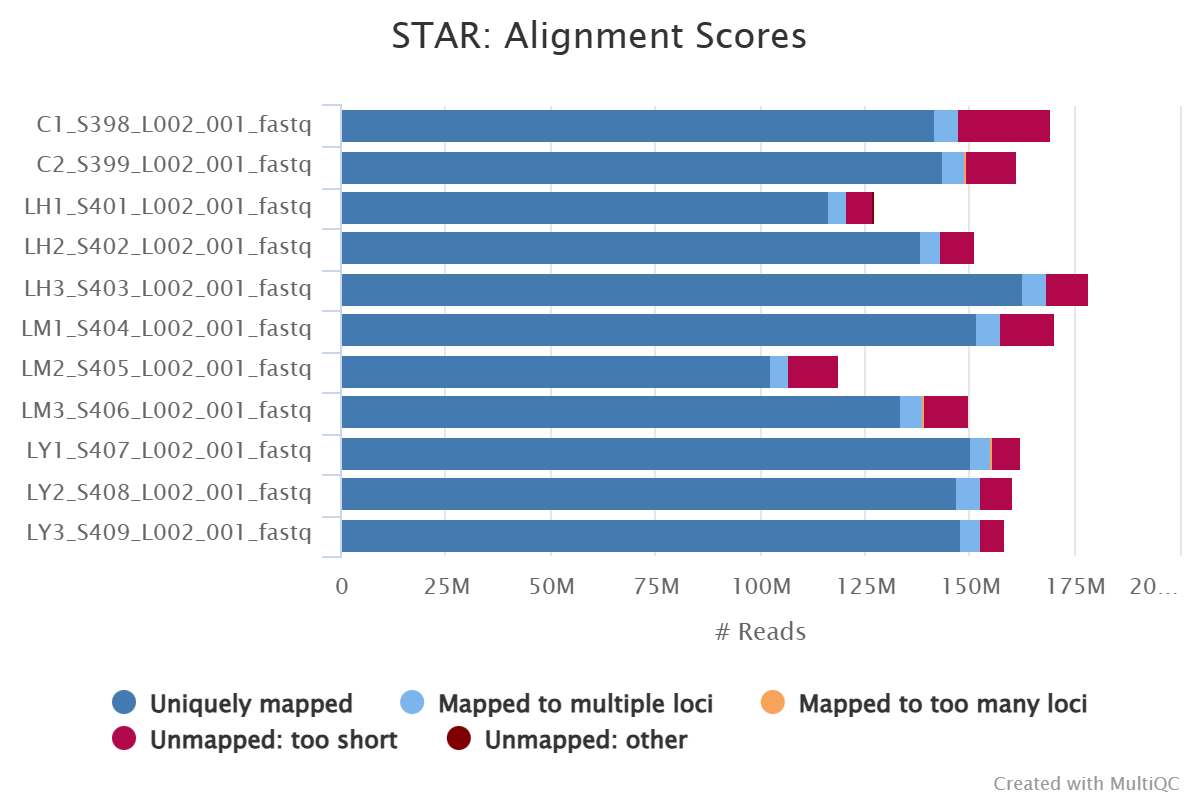

Supplement: Supplementary file 2 — Supplementary Material 2. [file 12864_2026_12791_MOESM2_ESM.png]
